# Towards automatic farrowing monitoring—A Noisy Student approach for improving detection performance of newborn piglets

**DOI:** 10.1371/journal.pone.0310818

**Published:** 2024-10-02

**Authors:** Martin Wutke, Clara Lensches, Ulrich Hartmann, Imke Traulsen

**Affiliations:** 1 Institute of Animal Breeding and Husbandry, Faculty of Agricultural and Nutritional Sciences, University of Kiel, Kiel, Germany; 2 Faculty of Agriculture, South Westphalia University of Applied Sciences, Soest, Germany; 3 Chamber of Agriculture Lower Saxony, Division Agriculture, Oldenburg, Germany; 4 Department of Animal Sciences, Georg-August University, Göttingen, Germany; Universidade do Porto Instituto de Biologia Molecular e Celular, PORTUGAL

## Abstract

Nowadays, video monitoring of farrowing and automatic video evaluation using Deep Learning have become increasingly important in farm animal science research and open up new possibilities for addressing specific research questions like the determination of husbandry relevant indicators. A robust detection performance of newborn piglets is essential for reliably monitoring the farrowing process and to access important information about the welfare status of the sow and piglets. Although object detection algorithms are increasingly being used in various scenarios in the field of livestock farming, their usability for detecting newborn piglets has so far been limited. Challenges such as frequent animal occlusions, high overlapping rates or strong heterogeneous animal postures increase the complexity and place new demands on the detection model. Typically, new data is manually annotated to improve model performance, but the annotation effort is expensive and time-consuming. To address this problem, we propose a Noisy Student approach to automatically generate annotation information and train an improved piglet detection model. By using a teacher-student model relationship we transform the image structure and generate pseudo-labels for the object classes piglet and tail. As a result, we improve the initial detection performance of the teacher model from 0.561, 0.838, 0.672 to 0.901, 0.944, 0.922 for the performance metrics Recall, Precision and F1-score, respectively. The results of this study can be used in two ways. Firstly, the results contribute directly to the improvement of piglet detection in the context of birth monitoring systems and the evaluation of the farrowing progress. Secondly, the approach presented can be transferred to other research questions and species, thereby reducing the problem of cost-intensive annotation processes and increase training efficiency. In addition, we provide a unique dataset for the detection and evaluation of newborn piglets and sow body parts to support researchers in the task of monitoring the farrowing process.

## Section 1: Introduction

Monitoring farrowing in livestock farming is of crucial importance to gain deeper insights into the physiological nature and complex behavioral characteristics of the birth process and to access critical information like the onset of farrowing, the specific time intervals between consecutive birth events or the overall farrowing duration [1]. As the intensity of pig production systems has increased over the years, new challenges in farrowing and concerns about reduced animal welfare have emerged [2, 3]. Thus, space limitations can lead to an increased stress level and a reduced expression of natural behaviors [4], which is crucial for an adequate welfare assessment [5, 6]. In addition, the challenge of adequately monitoring the status of sows increases as the animal-to-staff ratio increases as well [7], making the early detection of problems during farrowing more difficult. As both direct as well as manual video-based observation require an extensive amount of monetary and time-related resources, innovative methods from the field of computer vision (CV) and deep learning (DL) offer the potential to automatically monitor the animals and to provide important information about their welfare status and the overall farrowing process. Here, previous studies have shown promising results in the course of automatically monitoring different production stages within pig farming systems for example to analyze the behavior of prepartum sows [8, 9], to access essential welfare indicators like the development of the animal’s body mass [10–14] or the detection of individual pigs [15].

Although various studies have successfully shown the scale of DL and CV for the extraction of husbandry-related information, most CV approaches have been designed and conducted based on a large number of manually annotated images. For example, Ho et al. (2021) implemented a CV-based framework for monitoring the lactation frequency of sows by detecting and tracking newborn piglets [16]. In the course of their study the authors manually annotated 1611 images containing a varying number of piglets. Furthermore, Liu et al. (2023) increased the usability of a regular bounding box detection by determining the animals orientation using rotated bounding boxes for which 38699 pig instances within 3123 images had to be manually annotated [17]. Although it is well known that the underlying annotated data forms the basis and backbone of every supervised machine learning algorithm, the manual annotation process limits the power and generalizability of these methods for future research and the transferability into practice.

To achieve a high model performance, sufficient high-quality labeled data samples are required [18]. Particularly, in situations such as the application of early warning systems, where accurate object detection results are crucial for avoiding false alarms, a high data quality is necessary. However, the corresponding manual annotation process is cumbersome, error prone and cost intensive [19, 20]. Despite the promising results of recent DL-based object detection studies, the detection of dense target objects like newborn piglets is challenging for many state-of-the-art detection frameworks [21], where the number, shape, position or posture of the piglets can vary significantly. In addition, free farrowing systems are increasingly being used to enhance animal welfare, but these systems increase the complexity to automatically monitor the entire pen due to the potentially higher risk of piglet occlusion and a larger variation of possible sow postures.

In recent years, the importance of object detection in livestock husbandry has increased significantly. Here, object detection refers to the task of locating multiple objects within an image, each belonging to a distinct category of interest [22]. Especially in the scientific community, object detection is more and more applied to support researchers in tackling various research questions. For example, Qiao et al. (2023) use object detection to determine individual body parts of cattle, which, in the authors’ view, would improve automatic phenotyping or enhance the evaluation process of specific behavioral situations [23]. Küster et al. (2021) also use object detection in the form of a bounding box annotation to quantify and evaluate the frequency of interactions between sows and their respective pen facilities [9]. Another promising area of application that is currently being actively researched is the use of object tracking algorithms to evaluate animal behavior over time. Current state-of-the-art tracking applications incorporate convolutional neural networks (CNNs) and a tracking by detection approach where object detection provides the basic information [24–28]. Although the aforementioned studies show the potential of object detection algorithms to address specific problem scenarios, the applicability of most methods within a commercial setting is still challenging. On the one hand, most approaches yield satisfying results within the boundaries of their respective study design, but their performances decreases significantly under new conditions due to a lack of model generalizability. On the other hand, the rising cost in human labor [29] and the complex nature of animal behavior increases the need for sufficient annotated data samples, making manual model adjustments inefficient, cumbersome and cost intensive.

One way of mitigating the labor intensive process of creating large-scaled datasets is to deploy a self-training method like the Noisy Student Training algorithm which was originally proposed by Xie et al. in 2020 for image classification [30]. The general idea of the Noisy Student method is that a supervised model is trained as an initial teacher model entirely on manually labeled data samples and is then applied on new, unseen samples to create pseudo-labels [31]. After that a second model, referred to as the student model, is then trained using the large-scaled pseudo-annotations as well as the the manual annotations. During the student model training additional noise, such as random data augmentation, stochastic depth and random dropout is introduced to increase the generalization performance of the student compared to the teacher [29].

Despite the reported significant performance improvements by Xie et al. (2020), the Noisy Student concept has so far found very little recognition in agricultural research. As one of the few studies utilizing this method Duong et al. (2024) compared the Noisy Student concept for the multi-class classification of weed images to other transfer learning strategies [32]. Emphasizing the complexity of real-world scenarios, the Noisy Student method achieved the highest classification performance with an accuracy score of 99.26%. In addition, Keh (2020) used the Noisy Student method to compare different CNN architectures like VGG16, ResNet101, and DenseNet161 to classify plant pathogens in order to address the problem of lost crops due to pests and other pathogens [31]. As a result, they identified the EfficientNet structure to be the most efficient model for the given task and reported that applying the Noisy Student approach significantly improved the robustness and convergence rate of the model during training.

As Liu et al. (2021) point out, a large share of previous studies have focused on transfer learning methods for image classification tasks, while object detection tasks are underrepresented due to a higher complexity of instance annotation [33]. In this work we aim to address this limitation and additionally leverage the Noisy Student method to improve the detection performance of newborn piglets. More specifically, the contribution of this work is threefold. First, we extent the scope of the Noisy Student method by introducing a novel image transformation based on the pseudo-labels of the teacher model for subsequently training the student model. This transformation allows us to reduce the possibility space of the detection model and to focus on the target class in the form of newborn piglets. To the best of our knowledge, this is first study combining the self-learning Noisy Student approach with an image transformation by reducing the observable space to a restricted target area. As we demonstrate, this transformation step reduces the influence of complex pen environments and increases the detection efficiency of newborn piglets without the need to monitor the entire farrowing pen. It is therefore possible in subsequent studies to change this target area to any image area in order to focus on different target classes and to address alternative research questions. Second, there are currently hardly any publicly available datasets for newborn piglet detection which limits the power of existing machine learning methods for object detection. To address this limitation, we created a novel manually annotated dataset for the detection of sow body parts and newborn piglets and made this dataset publicly available. Third, automatic piglet detection is an intensively studied field and an important step for the implementation of early warning systems and automatic monitoring solutions. To the best of our knowledge this is the first study in the area of livestock research to leverage and extend the self-learning Noisy Student Training concept for the detection of newborn piglets. With this work, we aim to demonstrate the potential of our approach which can be used to address various research questions within the livestock domain like behavioral analysis, automatic phenotyping or the detection of agonistic anomalies.

This work is structured as follows. Section 2 provides a description of the data used for this analysis and illustrates the methodical foundation of the Noisy Student training rationale as well as the process of the model evaluation applied in this study. The results of the model comparison in detecting newborn piglets between the teacher model, trained on manually annotated images, and the student model, trained on automatically generated pseudo-labels, are presented and discussed in Section 3. Furhermore, a model comparison for different dataset sizes in presented. Section 4 concludes this work and provides an outlook for future research.

## Section 2: Materials and methods

### Section 2.1: Data acquisition and processing

In this study an extensive video dataset was collected between March 2021 and September 2023 as part of the collaborative project DigiSchwein (funding code: 28DE109G18) at the research farm of the Chamber of Agriculture Lower Saxony in Wehnen, Germany. In addition to other work packages of DigiSchwein that focus, for example, on the prevention of tail biting outbreaks or the analysis of nutrient flows, our aim is the development of a birth monitoring tool to assist farm staff by providing critical information about the health of the animals and the entire farrowing process. In the course of this study parts of the data have been utilized to develop and evaluate the proposed Noisy Student piglet detection model. For data acquisition, a static camera of the type AXIS M3206-LVE (Axis Communications AB, Lund, Sweden) was assembled 3m above the ground of eight farrowing pens. Over a period of 23 batches, each lasting 40 days, each camera recorded the farrowing process with a frame-per-second (FPS) rate of 20 frames and a display resolution of 1920*1080 pixels.

**Fig 1:**
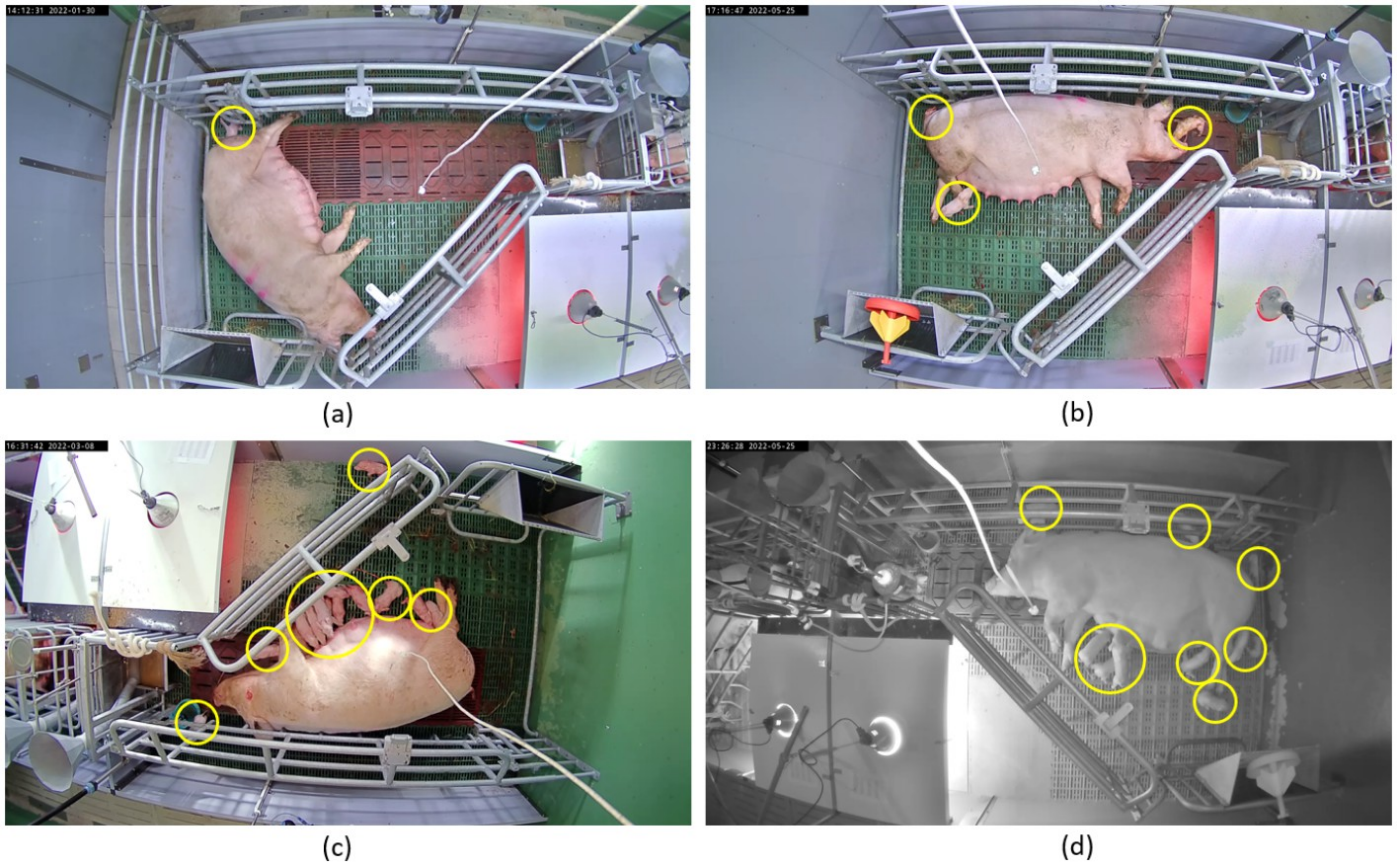
Example frames of different farrowing pens and a varying intensity of piglet occlusion. The occlusion can be caused by the pen infrastructure, the sow or piglets. The number of piglets (yellow circles) ranges from one and three piglets in (a) and (b) to twelve and ten piglets in (c) and (d). Frames (a—c) have been captured at daylight, while frame (d) has been taken at night-time.

The dataset used for this study is comprised of video sequences containing both situations before farrowing, in which only the sow is visible, as well as situations during and after farrowing where both the sow and newborn piglets are visible. Both day and night recordings with varying lighting conditions have been used. In order to avoid a potential bias of the detection models for different color settings, each video frame has been grayscaled prior to the process of model training and evaluation [34, 35]. Furthermore, to increase computational efficiency, the original image dimension was downscaled to 640*640 pixel and used as the input dimension for the training process. Fig 1 exemplarily shows four video frames at different lighting conditions and a varying number of newborn piglets, which have been manually highlighted by yellow circles.

As can be seen in Fig 1 monitoring the entire farrowing pen inevitably leads to a high number of occluded piglets, depending on the length of the observation time, the camera’s recording angle, the equipment in the pen, the posture of the sow or the number of piglets. Therefore, the decision to monitor the entire pen or only parts of it depends on the research question and the aim of the study. Since one of the main tasks of the collaborative project DigiSchwein is to access critical farrowing information about newborn piglets like individual birth intervals, a close observation of a restricted birth area within the video frame is desired without the need of monitoring the entire pen, thus reducing the complexity of the scene.

**Fig 2:**
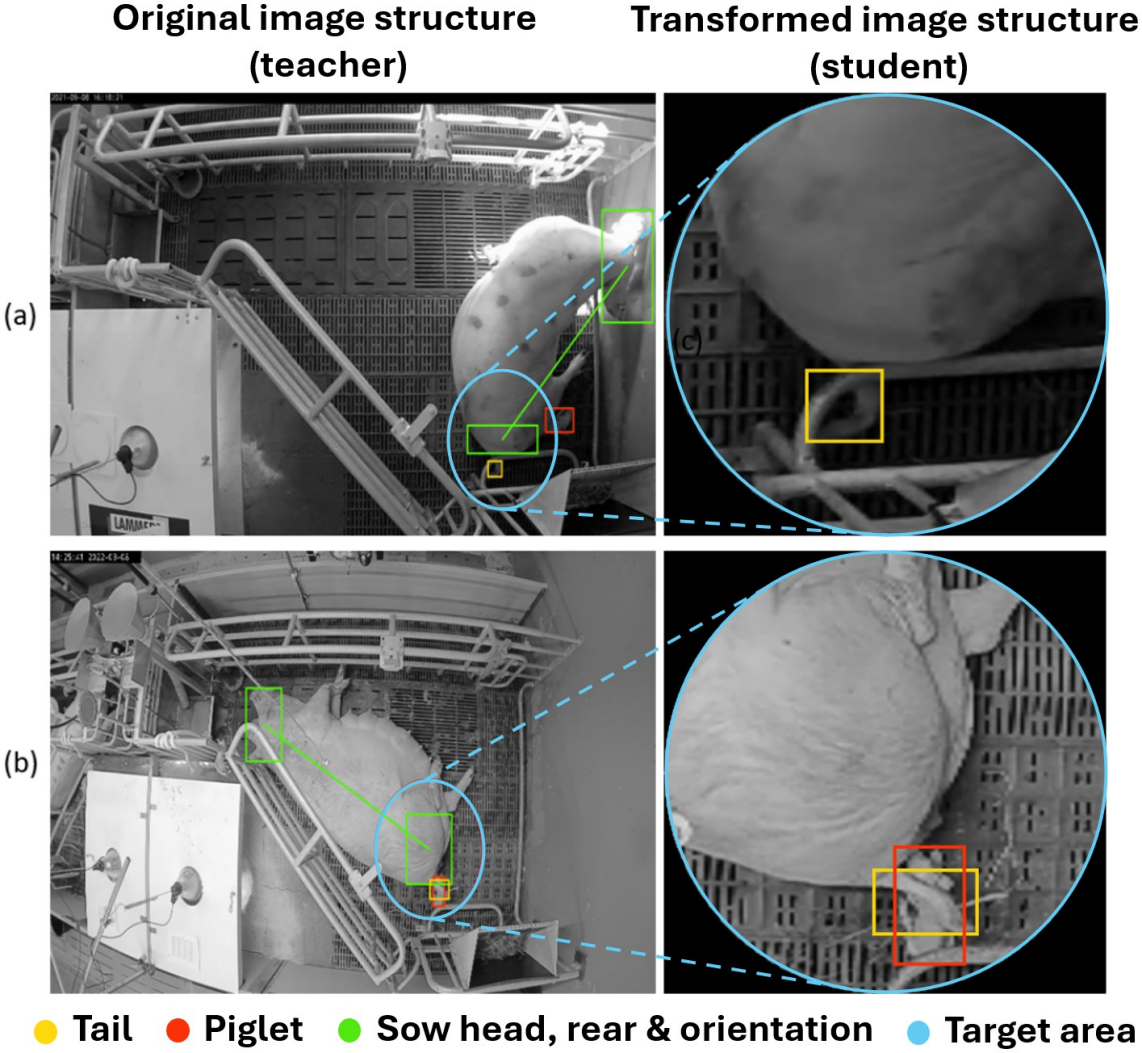
Example of the image structure transformation. The image dataset of the teacher model consists of images with four distinct object classes, namely the head of the sow (green box), the rear region of the sow (green box), the sows tail (yellow box) and newborn piglets (red box). The head and tail information have been used to compute the orientation of the sow (green connection line) and to compute the restricted birth region (blue circle). For the generation of the target image structure of the student model only the restricted birth region has been used to reduce the possibility space to only two object classes, tail and piglet. All subfigures in this illustration are daylight recordings that have been converted to grayscale images for further processing.

In order to generate the required image structure, a Noisy Student approach is chosen, in which an initial object detection model (hereafter referred to as the teacher model) first detects the head and rear region of the sow and then uses these information to select a distinct birthing area at the rear of the sow. This image area is then cropped and rescaled to a pixel dimension of 640*640 pixel. The subsequent detection models are trained and evaluated on this target image structure. An illustration of the original image and the final target image structure is provided in Fig 2. Here, two example frames from the teacher model training set have been selected and annotated using a bounding box annotation for the object classes *head*, *rear*, *tail* and *piglet*. A detailed description of the training process for both the teacher and the student model is given in Section 2.2.

In the course of this study two types of datasets have been employed. To train the teacher model, 1100 images of the whole farrowing pen have been manually labeled using a bounding box annotation for the above mentioned object classes (*head*, *rear*, *tail* and *piglet*). For the process of image annotation the software LabelMe (Version 5.2.1) was used [36]. In contrast, the training process of the student model is carried out using the target image dataset which consists of 9,800 images, that have been automatically annotated using the Noisy Student approach (Section 2.2). Since the student model is designed to monitor the target area, the possibility space was further reduced to only two object classes, namely *tail* and *piglet*. Additionally, we performed simple image augmentations in the form of horizontal and vertical flips for both datasets to increase the dataset size by factor four. To stimulate further research, we made the manually annotated dataset for the teacher model publicly available [37].

### Section 2.2: Noisy student training

**Fig 3:**
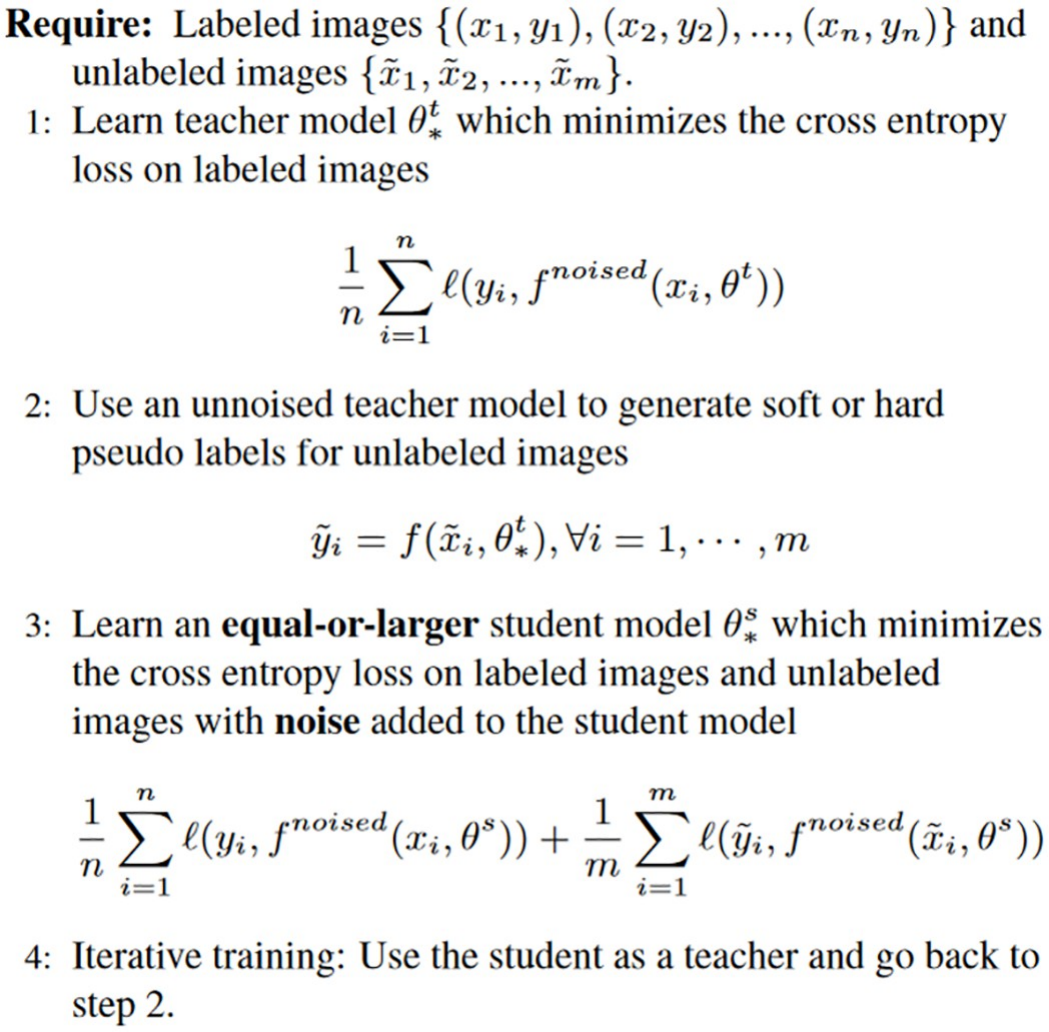
Illustration of the Noisy Student algorithm proposed by [30].

The concept of Noisy Student training thematically belongs to the area of SSL and the theoretical foundation of a self-training framework was already mentioned in 1965 by Henry Scudder [38]. In recent years, SSL-based object detection has shown great potential due to its straightforward approach and reduced dependency on resource-intensive annotations [39–41]. Although Noisy Student training has been extensively used in the field of computational linguistics like speech recognition or analysis (e.g. [42–46]), it is nowadays increasingly being used in other disciplines as well. In the area of CV Xie et al. (2020) [30] showed that the addition of noise during the model training has a positive effect on the quality of the pseudo-label generation and the performance of the subsequent student model. Furthermore, using Noisy Student as a self-learning approach, they have been able to improve the classification performance on the well known ImageNet dataset [47] by two percent compared to state-of-the-art models at that time. The Noisy Student algorithm is illustrated in Fig 3.

**Fig 4:**
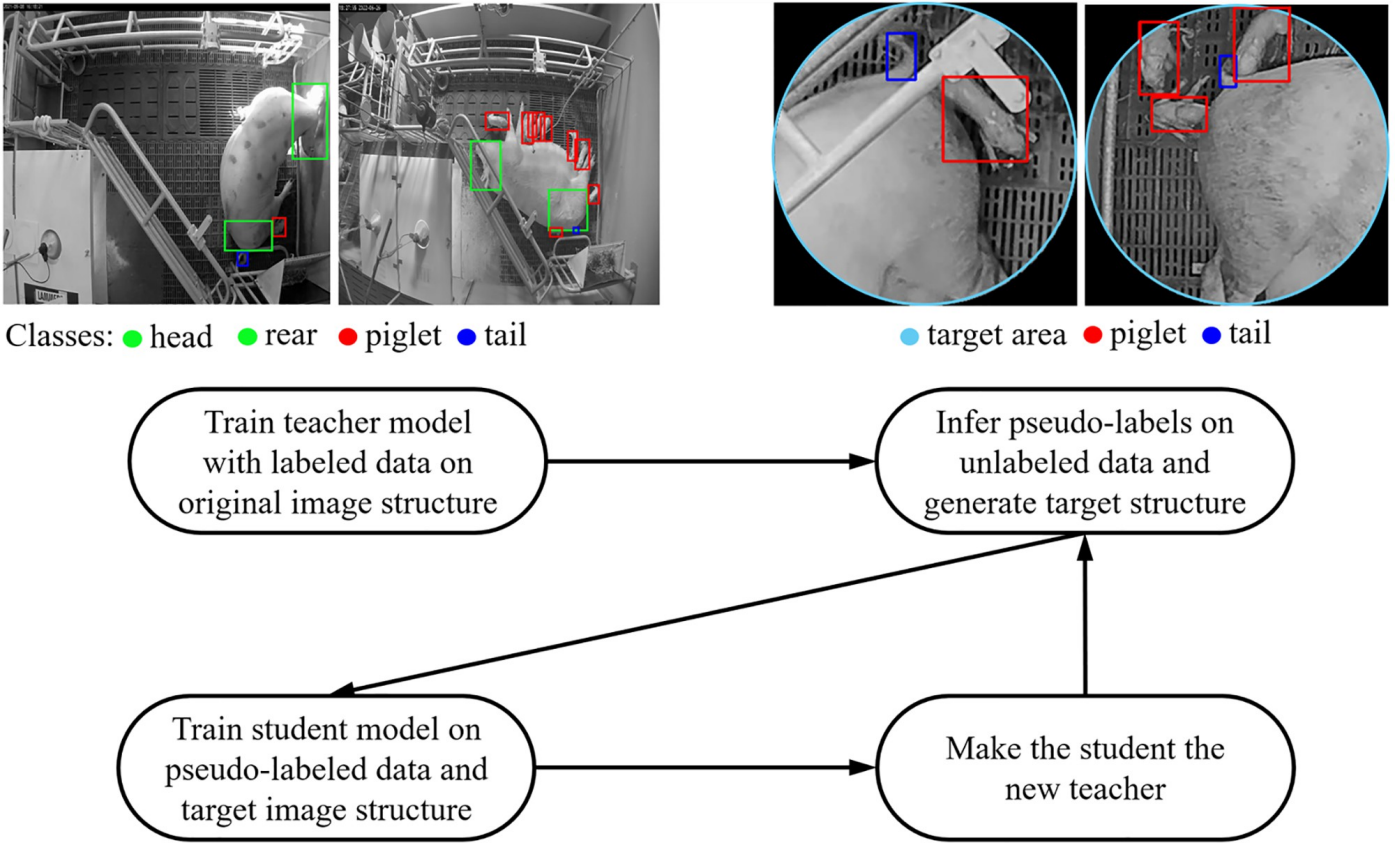
Overview of the Noisy Student concept. While the teacher model was trained entirely on manually annotated images, we extended the Noisy Student concept and trained the student model only on the new image structure based on the pseudo-labels within the designated target area. All images shown in this figure are from the teacher and student training dataset. All subfigures in this illustration are daylight recordings that have been converted to grayscale images for further processing. This figure is based on [30].

In the Noisy Student setting an initial teacher model is first trained on a small set of labeled information and is then applied to generate pseudo-labels for a larger set of unlabeled data [48]. After that, a second model, denoted as the student model, is trained on the pseudo-labeled data and is subjected to noise in the form of dropout and random augmentation. In an iterative manner, the student is then considered as the new teacher to obtain a more robust student model. Finally, this training procedure is repeated until the student’s performance is satisfactory for a given task [49]. Fig 4 illustrates the basic idea of the Noisy Student method applied in this work.

As can be seen in Figs 2 and 4, we extended the Noisy Student method by introducing a novel image transformation and the usage of two distinct image structures. The teacher model was trained on the original images which covered the whole farrowing pen but were therefore more susceptible to object occlusion and misclassification. In contrast, in order to improve the detection rate of newborn piglets we focused only on the designated target area and generated the pseudo-labels for the student model by only using high confidence detections above the 0.9 confidence threshold. Additionally, as suggested by previous studies [50–52] we manually checked the generated pseudo-labels and verified the correctness of the class assignment and bounding box localization. Image instances containing incorrectly labeled information have been removed from the training set of the student model accordingly.

As it was shown by [30] the inclusion of additional noise to the training process of the student model like data augmentation and random dropout can improve the performance and generalization ability of the student. Therefore, we adopted this strategy and additionally used random dropout [53] and mixup augmentation [54], each with a rate of 0.5 only for the student training. The general purpose of data augmentation is to train a model on similar but different instances that are present in the training data [55]. The mixup augmentation was originally proposed for image classification tasks and proved successful in reducing adversarial perturbation in the network structure [54]. The basic idea is to linearly interpolate multiple image samples and their corresponding labels to generate new training instances [56]. As an extension, Zhang et al. (2019) successfully demonstrated the implementation of a mixup augmentation for the task of object detection without adding additional overhead costs during inference [57]. By merging multiple images with their corresponding labels they tested various mixup sampling distributions and reported a final improvement of up to 5% absolute precision compared to state-of-the-art baselines at that time.

### Section 2.3: Model training and evaluation

In the course of this study, all detection models have been trained using the YOLOv8-Architecture which has been published by Ultralytics in 2023 [58]. YOLOv8 is a state-of-the-art object detection model which is able to handle the multiscale nature of objects [59]. Since this study aims at leveraging the Noisy Student Training concept for improving detection performance of newborn piglets, the largest available model version (YOLOv8x) was selected. While the first version of YOLO (You Only Look Once) was developed by Redmon et al. in 2016 [60] as an anchor-based method, YOLOv8 follows an anchor-free approach which leads to a reduced number of box predictions and improves processing speed [61, 62].

Both the teacher and the student model have been trained for 300 iterations with a batch size of 16 images per batch and an input image dimension of 640*640. The decision to train the models for 300 training iterations was determined empirically, as no significant improvements were achieved with a higher number of iterations. We followed previous studies and used the Stochastic Gradient Descent optimizer with a learning rate of 0.01 [63–66] to train all models. The model training was carried out on a workstation equipped with two Intel Xeon Gold 6230 CPUs, 1024 GB RAM, and a Nvidia Quadro RTX 8000. The models have been implemented using the programming language Python (version 3.9), the deep learning framework Pytorch (version 2.0.1) and Ubuntu 22.04.3 as the operating system. During the training process no signs of overfitting have been detected for both the teacher and the student model.

In order to access the overall detection performance and to be able to adequately compare both the teacher and the student model, we followed previous studies and evaluated each model based on the well-known performance metrics *Recall*, *Precision* and *F1-score* [67–70]. Here, we first determined the number of *True Positives* (*TP*), *False Positives* (*FP*), and *False Negatives* (*FN*) over all test images. The evaluation metrics are defined as follows:

$$\begin{array}{c} Recall=\frac{TP}{TP+FN} \end{array}$$ (1)

$$\begin{array}{c} Precision=\frac{TP}{TP+FP} \end{array}$$ (2)

$$\begin{array}{c} F1=\frac{2TP}{2TP+FP+FN} \end{array}$$ (3)

For the evaluation process we generated a distinct evaluation dataset comprised of 650 randomly selected images and ground truth annotations. Since the student model is trained on a reduced number of classes, we used the manual annotations of the sows head and rear region to generate the required target image structure. For the teacher model, we only used the detection results within the target region and compared these results with the detection results of the student model (see Fig 2). Table 1 provides an overview of the class distribution of the training and evaluation sets.

**Table 1:** Distribution of target classes in absolute (percentage) frequencies for the training data of the teacher and student model and for the evaluation dataset.

Number of	Teacher Train	Student Train	Evaluation
Dataset			
Head	4,519 (32.09%)	-	-
Rear	4,530 (32.17%)	-	-
Sow tail	2,685 (19.06%)	28,761 (56.69%)	256 (27.01%)
Piglet	2,349 (16.68%)	21,974 (43.31%)	692 (72.99%)
Total	14,083 (100%)	50,737 (100%)	948 (100%)

## Section 3: Results and discussion

In the following section, the results of the detection evaluation are presented and critically discussed on the basis of the methodology described in Section 2. Existing challenges are addressed in detail and design choices are motivated.

To assess the suitability of the proposed Noisy Student approach for improving detection performance of newborn piglets, we were interested in the performance of the student model, trained on pseudo-annotated data samples, against a model trained on manually annotated data. Since the initial teacher model was trained on 1100 human annotated images, we used this model as a proxy for a manual labeling approach and performed the comparison between the final student model and the initial teacher model. For the creation of the evaluation dataset, the two target classes *piglet* and *tail* as well as the body parts *head* and *rear* have been manually annotated in 650 test images. The evaluation dataset is made publicly available [37].

To ensure an objective assessment, both models were evaluated only on the basis of their detection performance for the piglet and tail object classes in the target area. For the teacher model, only the detection results within the defined birth area were included. The results of the model evaluation are shown in Table 2, where the detection results are presented separately for each target class as well as in an aggregated form, in which both target classes have been consolidated prior to the calculation of the evaluation metrics.

**Table 2:** Results of the detection evaluation for the teacher and the student model on the evaluation set. Each model performance is listed both as a total result and differentiated by the target object class.

*Model*	*Class*	*# TP*	*# FP*	*# FN*	*Recall*	*Precision*	*F1*
Teacher	Piglet	441	4	249	0.639	0.991	0.777
	Sow tail	90	99	166	0.352	0.476	0.404
	**Total**	**531**	**103**	**415**	**0.561**	**0.838**	**0.672**
Student	Piglet	666	25	24	0.965	0.964	0.965
	Sow tail	186	26	70	0.727	0.877	0.795
	**Total**	**852**	**51**	**94**	**0.901**	**0.944**	**0.922**

As can be seen in Table 2, focusing on the consolidated results both models achieved very good Precision values, indicating that most objects detected by the models have been correctly localized and classified. With a value of 0.944, the student model achieved a value almost ten percent higher than the teacher model with a value of 0.838. In terms of Recall, the difference between the teacher and student model is greater, with the student model achieving a score of 0.901 compared to the teacher model with a score of 0.561. Since the Recall is the proportion of true positive detections over all positive ground truth information, it reflects a detectors ability of detecting an object of interest, without taking into account the correctness of the detection [71]. Since both models have high Precision values, the difference in Recall can be explained by the higher number of undetected objects by the teacher model, which is reflected in the form of higher FN values. Subsequently, given that the F1-score is the harmonic mean of Recall and Precision, the teacher model’s lower Recall is responsible for its F1-score of 0.672, in contrast to the student’s higher F1-score of 0.922.

**Fig 5:**
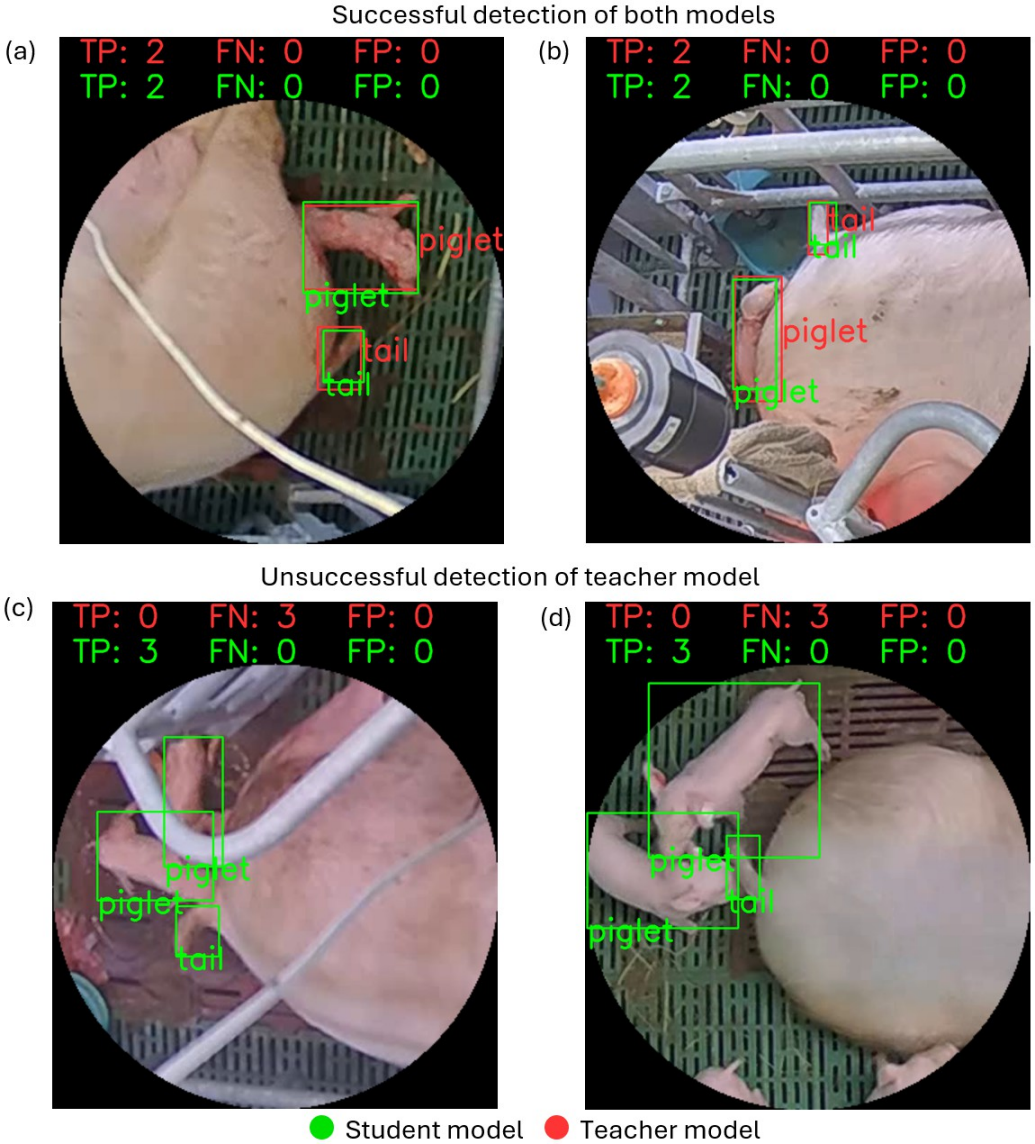
Visualized results of the model comparison. Samples from the evaluation set showing the detection results from the teacher model (red bounding box) and the student model (green bounding box). (a-b): In cases of a good spatial separation between the objects, both detection models achieve satisfying results in detecting the piglet and tail classes. (c-d): In situations of overlaying objects or close proximity the detection performance of the teacher model decreases. For the interpretation of the color information in this illustration, the reader is referred to the web version of this article.

From a more differentiated perspective, it can be seen that both models show a better performance in detecting the piglet class compared to the tail class. In line with the previous results, the student model with a Recall score of 0.965 for the piglet class exceeds the performance of the teacher model, which achieved a Recall value 0.639. For the tail class the student model achieved a Recall 0.727 whereas the teacher achieved a value of 0.352. Fig 5 provides sample images from the manually generated evaluation set in which the results of the teacher as well as of the student model have been visualized.

By visualizing samples of the detection results it can be seen that the low detection performance of the teacher model for both the tail as well as for the piglet classes occurs mainly in situations in which the object to be detected is in close proximity to another target object. The proximity effect is observable in the student model as well, albeit to a lesser extent. However, the problem of close proximity and object overlays is not exclusive for this study and is frequently reported to be one of the main obstacles in object detection studies as well as video object tracking applications [34, 72–74]. In cases of good separation both models are able to successfully localize and classify most of the tail objects. Since the motivation for adding the tail class was to reduce the risk of false detection and a potential mixup with the piglet class, it was not the aim of this study to optimize detection performance for this particular class. In contrast, the situation is different when looking at the piglet detection performance. As the correct detection of newborn piglets is essential in the automatic assessment of the farrowing process, a robust model performance is required. Here, it can be seen that in many cases the close proximity of multiple piglets poses a major challenge for the teacher model.

**Fig 6:**
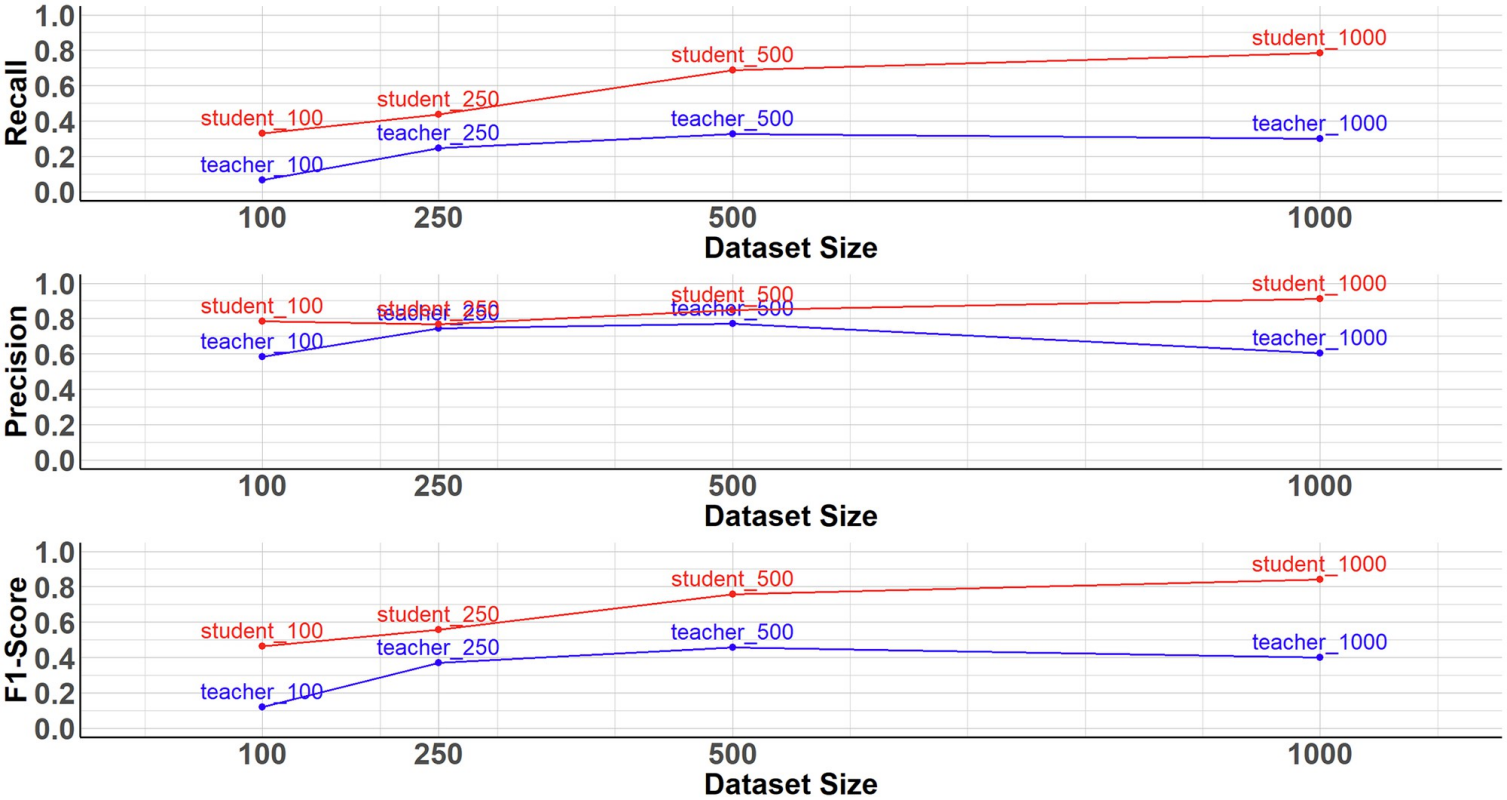
Results of the model comparison with different dataset sizes. When comparing different dataset sizes, the Noisy Student model (blue line) trained on the transformed target images shows a superior detection performance compared to the teacher model (red line).

To additionally investigate the potential of the presented extended Noisy Student method to improve the generalization properties, we performed another model comparison with different data set sizes. Here, we have created a subset of fixed size for each model type by randomly selecting an image from the respective training set. The data set sizes 100, 250, 500 and 1000 training images were defined as the object of investigation. A new teacher and student model was then trained for each level of the dataset size and evaluated on the evaluation data set presented above. The results of this second model comparison step are shown in Table 3 and illustrated in Fig 6.

**Table 3:** Results of the model comparison with different dataset sizes.

*Model*	*DS Size*	*Recall*	*Precision*	*F1-Score*
Teacher_100	100	0.067	0.583	0.120
Student_100	100	0.331	0.784	0.465
Teacher_250	250	0.245	0.746	0.369
Student_250	250	0.438	0.767	0.557
Teacher_500	500	0.326	0.770	0.458
Student_500	500	0.687	0.849	0.759
Teacher_1000	1000	0.300	0.604	0.401
Student_1000	1000	0.780	0.910	0.842

As Table 3 and Fig 6 show, the performance of the student model outperforms the teacher model for each level of data set size and each of the three evaluation metrics Recall, Precision and F1-score. This indicates the superior detection and generalization ability of the student model and demonstrates the positive effect of the proposed image transformation.

As this study proposes a complexity reduction of the image scene by transforming the original image structure for training the subsequent student model, the corresponding design choices have been motivated by the increasing need for monitoring free farrowing environments and precisely monitoring the birth process. With enhanced welfare requirements on the one hand and the current trend of increasing livestock intensity on the other, free farrowing systems are gaining in importance but animal observation pose greater challenges. Although earlier studies adopted a strategy of automatically monitoring the entire pen area [16, 75, 76], this approach leads to increased computationally complexity due to the problem of blind spots and occluded piglets. The problem of object occlusion is currently one of the biggest challenges in CV systems and can influence detection results and hinder the assessment of the farrowing status [77–79]. Therefore, to reduce the need to monitor the entire pen and to increase the potential generalization ability of the student model to multiple pen structures, piglet detection was focused on the selected target area, which appears to be beneficial for the detection performance of the student.

In recent years, the method of object detection has become increasingly popular and is nowadays used for a variety of tasks in academic research. However, current approaches in the agricultural context mainly focus on fully-supervised learning methods, where data annotation is challenging and time consuming [80]. Although self-learning methods are also more frequently used, they are still underrepresented. When compared with other self-learning studies for object detection tasks our findings are in line with previous results, reporting an increased performance of the student model. For example, Zhang et al. (2023) focused on the knowledge transfer from the teacher to the student using knowledge distillation methods and reported an enhanced detection performance of the student as it learns to emphasize important pixels [81]. Similar findings are reported by Zhu et al. (2023) who applied a teacher-student relationship for the VisDrone [82] dataset which is mainly used for small object detection tasks [83]. In line with our results, the studies stated above report an improved detection rate of the student model for smaller and dense objects, which is beneficial for the detection and behavioral analysis of newborn piglets, as they are usually small in size and tend to appear in close proximity to each other.

The aim of this study was to improve the detection performance of newborn piglets by proposing an novel extension of the Noisy Student self-learning framework combined with the usage of a distinct key focus in the form of the selected target area. Furthermore, we wanted to address the limitation of the cumbersome annotation process of supervised object detection methods in the field of animal research. To the best of our knowledge, this is the first application of the Noisy Student methodology in the area of livestock science. We demonstrate the improved detection performance of the student model which is not exclusively limited to the use case presented in this work. The selection of the target area enables the methodological transfer to other species, application disciplines and research questions. In the context of farrowing, the focus of the framework could be shifted so that the student is trained on a different target area selection, for example, to gain deeper insights into lactation behavior and sow-piglet interaction. Furthermore, in a broader research setting, the student-teacher relationship could be used to first detect larger objects like whole animal instances and then analyze key body parts or monitor specific areas of interest to address current research questions such as agonistic interactions or automatic phenotyping.

Considering the background of increasing animal welfare regulations and more open farrowing systems, it can be assumed that the number of unfixed sows and piglets to be monitored will increase in the long term. In Germany in particular, a maximum restraining period of five days is prescribed by law from 2036 [84]. A larger number of free-ranging animals generally increases the complexity and thus the required detection performance of the model used. Most established methodological approaches for object detection usually address such challenges by manually re-annotating more heterogeneous image data. The approach presented here contributes to this issue by decreasing the need to manually annotate new data samples by specifically defining target regions of interest that could provide automatically generated annotation information.

## Section 4: Conclusion

The presented study utilizes the innovative concept of Noisy Student training and leverages its characteristics to propose a novel approach for improving the early detection of newborn piglets within a free farrowing system. To the best of our knowledge this is the first application of the Noisy Student concept within the livestock domain. By using the self-learning paradigm, we were able to combine the distinct teacher-student model relationship with an image transformation technique to reduce the influence of the environment-specific pen infrastructure and the risk of object occlusion, thus demonstrating the applicability of our approach in a multi-pen environment. As a result, we could increase the detection performance from an initial Recall rate of 0.561 to 0.901 while at the same time improving the Precision from 0.838 to 0.944.

The findings of this study not only contribute to the current challenges of appropriately capturing critical information during farrowing, but can also be transferred to other species and application areas in livestock management. Furthermore, the utilization of the Noisy Student approach reduces the time and monetary costs inherent to manual image labeling, which usually acts as a limiting factor and weakens the potential of DL approaches.
